# Knowledge Discovery for Higher Education Student Retention Based on Data Mining: Machine Learning Algorithms and Case Study in Chile

**DOI:** 10.3390/e23040485

**Published:** 2021-04-20

**Authors:** Carlos A. Palacios, José A. Reyes-Suárez, Lorena A. Bearzotti, Víctor Leiva, Carolina Marchant

**Affiliations:** 1 Departamento de Obras Civiles, Universidad Católica del Maule, Talca 3480112, Chile; cpalacios@ucm.cl or; 2 Programa de Magíster en Gestión de Operaciones, Facultad de Ingeniería, Universidad de Talca, Curicó 3344158, Chile; 3 Departamento de Bioinformática, Facultad de Ingeniería, Universidad de Talca, Talca 3460000, Chile; jareyes@utalca.cl; 4 Escuela de Ingeniería en Transporte, Pontificia Universidad Católica de Valparaíso, Valparaíso 2362807, Chile; lorena.bearzotti@pucv.cl; 5 Escuela de Ingeniería Industrial, Pontificia Universidad Católica de Valparaíso, Valparaíso 2362807, Chile; 6 Facultad de Ciencias Básicas, Universidad Católica del Maule, Talca 3480112, Chile; cmarchant@ucm.cl; 7 ANID-Millennium Science Initiative Program-Millennium Nucleus Center for the Discovery of Structures in Complex Data, Santiago 7820244, Chile

**Keywords:** data analytics, databases, data science, Friedman test, socioeconomic index, university dropout

## Abstract

Data mining is employed to extract useful information and to detect patterns from often large data sets, closely related to knowledge discovery in databases and data science. In this investigation, we formulate models based on machine learning algorithms to extract relevant information predicting student retention at various levels, using higher education data and specifying the relevant variables involved in the modeling. Then, we utilize this information to help the process of knowledge discovery. We predict student retention at each of three levels during their first, second, and third years of study, obtaining models with an accuracy that exceeds 80% in all scenarios. These models allow us to adequately predict the level when dropout occurs. Among the machine learning algorithms used in this work are: decision trees, *k*-nearest neighbors, logistic regression, naive Bayes, random forest, and support vector machines, of which the random forest technique performs the best. We detect that secondary educational score and the community poverty index are important predictive variables, which have not been previously reported in educational studies of this type. The dropout assessment at various levels reported here is valid for higher education institutions around the world with similar conditions to the Chilean case, where dropout rates affect the efficiency of such institutions. Having the ability to predict dropout based on student’s data enables these institutions to take preventative measures, avoiding the dropouts. In the case study, balancing the majority and minority classes improves the performance of the algorithms.

## 1. Symbology, Introduction, and Bibliographical Review

In this section, abbreviations, acronyms, notations, and symbols used in our work are defined in Table 1. In addition, we provide here the introduction, the bibliographical review on the topic about related works, and an overview of the models utilized together with the description of the sections considered in this paper.

### 1.1. Abbreviations, Acronyms, Notations, and Symbols

Next, Table 1 presents the symbology considered in this paper to facilitate its reading.

### 1.2. Introduction

Data mining integrates modeling and data analytics. Although it is based on several disciplines, data mining differs from them in its orientation towards the end rather than towards the means to achieve it, feeding on all of these disciplines to extract patterns, describe trends, and predict behaviors, taking advantage of the information obtained from the data [1].

Data mining is only one stage, but the most important, in the process of knowledge discovery in databases (KDD). Note that KDD is defined as a non-trivial process to identify valid, novel, potentially useful, and ultimately understandable patterns in often large data sets, and to extract relevant information from available databases [2,3,4]; see more details about the KDD process in Section 2. This process consists of several phases and incorporates database techniques, machine learning (ML), statistics, artificial intelligence, and decision-making systems.

ML is the study of computer algorithms that improve automatically through experience and by the use of data (learning) [1]. Note that ML is seen as a part of artificial intelligence. ML and data mining use the same algorithms, but their process and usefulness are different. These algorithms can be supervised or unsupervised depending on whether we know the outcome or not, respectively. The main difference between data mining and ML is that, without human participation, data mining cannot work, but in ML, human effort is involved only at the moment when the algorithm is defined. Unlike data mining, in ML, the machine must automatically learn the parameters of the models from the data. Then, ML uses self-learning algorithms to improve its performance. In summary, ML is oriented towards the result, while data mining is oriented towards knowledge discovery. Some algorithmic techniques used in data mining and ML correspond to supervised techniques: classification and regression, decision trees (DT), ensemble models (EM), *k*-nearest neighbors (KNN), logistic regression (LR), naive Bayes (NB), random forest (RF), and support vector machines (SVM); or to unsupervised techniques: artificial neural networks (ANN), clustering (CLU), and correlation analysis [5,6]. Note that, in the case of ANN, they are also very popular supervised methods in data mining and ML.

### 1.3. Related Works

Around the world, student retention is an essential aspect of higher education (HE) institutions and involves university rankings, the reputation of the institution, and its financial wellbeing [7,8,9,10,11]. Thus, at-risk students should be identified prior to beginning their studies. Especially in developing countries, student retention has taken on much importance.

According to the official information of the Chilean government, the HE undergraduate enrollment increased from 165,000 students in 1980 to more than 1.2 million in 2018 [12,13]. This increased enrollment has enabled more students access to HE, particularly for those who are the first in their family to achieve a university education. Then, such an achievement involves an additional challenge for the HE institutions related to maintaining this new group of students in its initial study program, whose maintenance has become one of the priorities for the Chilean Ministry of Education [14]. However, data regarding the HE activities that involve student performance, particularly when associated with student’s background, is limited or not always publicly available. Student’s variables such as socio-economic and cultural status, family values, individual characteristics, and pre-college academic experience have a relevant influence on the student’s dropout [15,16]. In addition, there may be a lack of vocational orientation and poor academic performance [17]. All these variables, inherent to students, remain as important elements of the present study according to the information reported in [14].

Note that dropout rates are not equivalent among educational variables associated with units and areas. For example, the dropout rates for health and engineering careers in Chile are 11% and 44%, respectively. Thus, it is also necessary to introduce these educational variables along with student’s variables. Based on information from the Chilean Ministry of Education, more than 50% of the students who enroll in HE institutions do not complete the program in which they were initially enrolled. This results in high resource losses for the government and institutions, as well as decreased opportunities for students and their families. These opportunities, of course, can result in problems of low national productivity, as well as other negative effects. Therefore, both governments and HE institutions should be concerned about these problems due to such a loss of resources [14]. Similar statistics can be found in other countries as well. Thus, in Chile, it is important to formulate a predictive model for identifying what type of students tend to drop out of the HE institutions and the level of study at which dropout occurs. This identification can help the HE institutions to focus on resources to improve student’s conditions and implement actions to enhance retention rates.

The dropout problem remains not only in Chile, but also in other countries, which is increased due to the continuous growth of student enrollment in HE institutions. This problem directly affects graduation rates and a country’s growth, in addition to hurting HE institutions around the world, which results in the need to state new and better student retention solutions for these institutions. Note that data mining and ML techniques can be a suitable tool to formulate predictive models for student retention at various levels of HE institutions. These models are necessary and relevant.

Educational data mining (EDM) is an emerging scientific area concerned with applying data mining techniques to explore large-scale data from schools and universities in order to understand the context in which learning and other educational issues occur [18,19,20]. Applications of EDM were presented in [19] and developed between 1995 and 2005, in traditional educational institutions, which was complemented with a more recent study by the same authors [21]. These studies described the following eleven different areas of EDM application: (i) analysis and visualization of data, (ii) construction of didactic material, (iii) development of conceptual maps, (iv) detection of undesirable student behaviors, (v) feedback for support instructors, (vi) grouping of students, (vii) planning and programming, (viii) prediction of student performance, (ix) recommendations for students, (x) modeling of students, and (xi) analysis of social networks.

EDM is the application of data mining techniques to data from educational institutions to answer questions and to solve problems [22], discovering information hidden in these data. EDM can be applied to model student retention based on data from different educational institutions [15,21]. According to [20], EDM techniques are appropriate to identify students who may have problems or exhibit unusual behavior, such as academic failure, cheating, dropout, erroneous actions, low motivation, misuse, and playing games.

Various ML algorithms have been used to detect these students early in order to provide them with sufficient time and adequate support to prevent dropout. Different variables were considered in [7,8], such as academic, demographic, and financial factors, to detect dropout using and evaluating several ML algorithms, including ANN, DT, LR, and SVM. EDM techniques to predict attrition among electrical engineering students after they completed their first semester in The Netherlands were applied in [23]. Such an application employed DT, LR, and NB, with demographic, pre-college, and university academic characteristics being included as variables. EDM has also been used to predict student dropout of e-learning courses [24], comparing the performance of three different ML algorithms in terms of overall accuracy, precision, and sensitivity. A wide range of ML algorithms were contrasted in [25], developing a variable selection procedure for academic, demographic, and financial characteristics, with DT and NB presenting the best performance.

Several other techniques have been reported in the recent literature related to predicting student retention/dropout of different HE institutions around the world. Table 2 exhibits a comparison of these techniques. Clearly, attrition affects educational institutions worldwide, but there is no consensus on the variables to be considered in the ML algorithms employed among the different studies reviewed. Thus, EDM research needs to be developed and adjusted according to local conditions or the specific characteristics of HE institutions and countries.

Prior research on the use of EDM in Chile was reported in [26,42]. However, these studies are not conclusive in terms of retention patterns due to the limitations of the data used. Hence, to the best of our knowledge, there are no recent studies of EDM in Chile that provide predictive models for student retention at various levels of HE institutions based on modern data mining and ML techniques. Therefore, the objectives of this study are: (i) to formulate EDM models based on ML algorithms for extracting relevant information with appropriate educational data in any HE institution with similar conditions to the Chilean case; and (ii) to utilize this information to help the KDD process.

### 1.4. Models and Description of Sections

**Figure 1:**
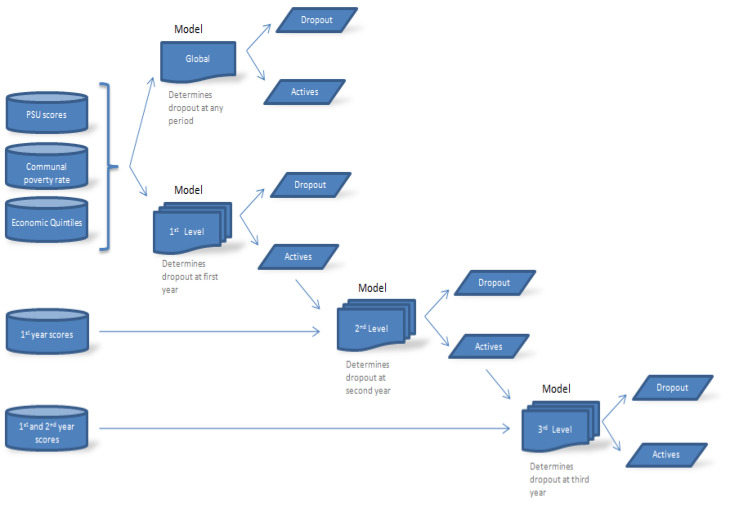
Scheme of the four different models proposed to predict student retention/dropout, where PSU indicates the university selection test.

Four models are proposed in this research as schematized in Figure 1. From this figure, the first one is a global model, which is proposed to predict the student retention regardless of the year (level) when student dropout occurred. The second model is formulated to predict the retention of freshmen students (considering who dropped out of their studies during the first year). The third and fourth models allow us to predict student retention during the second and third year of study, respectively, different from the first and second models because they incorporate university grades. A group of ML algorithms is applied to describe each model, whereas their performances are compared at each level in terms of accuracy and precision, as well as false positive (FP) and true positive (TP) rates. An unpublished real educational data set is used as a case study based on enrollment records from the Catholic University of Maule (Universidad Católica del Maule in Spanish) (UCM, http://www.ucm.cl (accessed on 15 April 2021)), a Chilean HE institution located in Talca, a city 253 km south of Santiago, the capital city of Chile. These data cannot be shared publicly as they contain potentially identifiable or confidential student information.

The rest of this paper is organized as follows. In Section 2, we present the methodology to be used. Section 3 applies the methodology to the Chilean real educational data. In this section, we provide an algorithm that summarizes the methodology proposed in our research. Some conclusions of the research are provided in Section 4. In this section, we also discuss knowledge discovery indicating the reasons for dropping out of studies beyond the results of the ML algorithms. In addition, we give some ideas about future research.

## 2. Methodology

In this section, we present the methodology to be used including its contextualization, and the steps of data selection, preprocessing, transformation, data mining/ML algorithms, and interpretation/evaluation.

### 2.1. Contextualization

**Figure 2:**
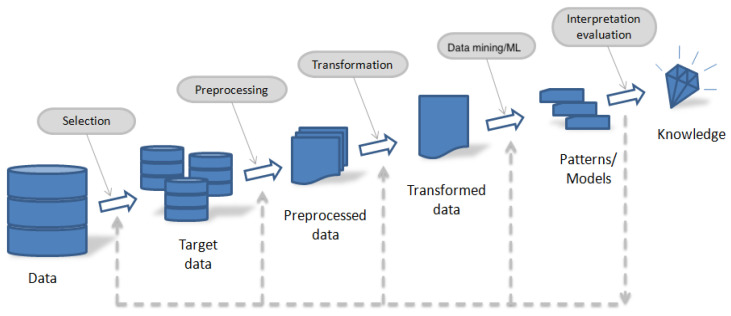
Scheme of the KDD methodology.

This study is based on the KDD methodology, but modified to formulate a model according to Figure 1. The KDD methodology includes an iterative and interactive process where subject’s experience is combined with a variety of analysis techniques including ML algorithms for pattern recognition and modeling development. Figure 2 exhibits a diagram of the KDD methodology, which consists of the following five ordered steps:

(i) Data selection,
(ii) Preprocessing,
(iii) Transformation,
(iv) Data mining/ML algorithms, and
(v) Interpretation/evaluation [43].

All these steps are described in the context of the present study. The first three steps are related to how data are gathered and processed. The fourth step is explicitly associated with the data mining modeling that evaluates diverse ML algorithms. Then, an interpretation and evaluation procedure is addressed in order to complete the KDD methodology.

### 2.2. Data Selection

Sources for data selection can vary depending on the study carried out. The data type can be associated with quantitative or qualitative variables, where the qualitative case may contain nominal or ordinal scales. Once relevant data are selected according to the aim of data mining, their preprocessing should be pursued [44]. For the case study considered here, see Section 3.

### 2.3. Preprocessing and Transformation

The preprocessing step is performed in order to organize the selected data set into a manageable form, which is necessary for the subsequent phases of the KDD methodology. Researchers focus on identifying missing or noisy data in the entire collected data set to be removed or transformed into new data. These undesirable data are collected probably during the student registration process and exhibit inaccuracies when compared to different data sets. Then, the remaining (desirable) data and features are analyzed using an information gain (IG) procedure, which is a tool to measure the correlation of different variables with the target variable (student dropout in or case). IG is one of the simplest, fastest, and most accurate procedures to rank features, which is suitable and able to work with a high dimensionality of variables [45].

The transformation step involves the preliminary data processing and the generation of new variables from the existing ones. For example, several correlated variables are processed in order to generate a single feature that embodied them, as in the case of student stratification age when initiating studies. This stage focuses on the normalization of different features and data selected for the study in order to standardize all the data on similar scales, thus avoiding bias problems due to a broad range of values for certain variables.

### 2.4. Data Mining/ML Algorithms

The data mining/ML step establishes the modeling phase properly. Next, we provide some details of the ML algorithms used in Section 3 (case study).

**Decision** **trees:** This is a decision support technique that employs a DT-type model and its possible options, which include probabilities and their corresponding expected financial values. The DT technique has an algorithmic or sequential structure with nodes that contain conditional control aspects and branches that represent the response of each aspect. Specifically, the DT technique may be formulated as a combination of statistical and computational tools to categorize, describe, or generalize a data set. Each instance used in DT is collected in the form (X,Y), with *Y* being the dependent variable corresponding to the response or target that we are trying to explain, classify, or generalize by means of the feature (independent variable) *X*.

***k*-nearest** **neighbors:** This is a non-parametric technique utilized for regression or classification, as a simple algorithm that stores the current data and classifies new data based on distances. In regression or classification, the input is based on the *k* closest training instances in the space of the independent variable (feature), whereas the output depends on whether KNN is employed for regression or classification. Note that *k* is a previously fixed value. For regression, the output is the average of the *k*-nearest neighbors. For classification, the output is an object assigned to the class most common among its *k*-nearest neighbors. Specifically, suppose we have *n* pairs of data or instances $\left(x_{1},Y_{1}\right),\cdots ,\left(x_{n},Y_{n}\right)$, where *Y* is the class label of the feature *X*, so that $X|Y=c∼F_{c}$, for c=1,2 classes, where $F_{c}$ is a probability model and “∼” denotes “distributed as”. Given a norm ∥·∥ and a point *x*, let $\left(x_{\left(1\right)},Y_{\left(1\right)}\right),\cdots ,\left(x_{\left(n\right)},Y_{\left(n\right)}\right)$ be an ordering of the training instances such that $∥x_{\left(1\right)}-x∥\leq \cdots \leq ∥x_{\left(n\right)}-x∥$. Then, the *k* instances must be retained from the current data set closer to *x* and take their values of *Y*.

**Logistic** **regression:** This is a statistical modeling technique, which can be considered within the class of generalized linear models and is often used to describe a binary response variable by means of quantitative or qualitative independent variables (features). For example, for an LR model with one independent variable *X*, which may be continuous or binary, the general form of the log-odds is $l=\beta_{0}+\beta_{1}x$, where the coefficients $\beta_{0},\beta_{1}$ are the regression parameters and *x* is the observed value of *X*. Note that this is a log-linear model, so that the odds are the exponent $o=b^{\beta_{0}+\beta_{1}x}$, corresponding to a non-linear model, since the odds are a non-linear combination of the independent variable, where the base *b* is usually taken to be the exponential function. Then, the associated probability function of Y=1 is $p=P\left(Y=1\right)=\exp\left(\beta_{0}+\beta_{1}x\right)/\left(\exp\left(\beta_{0}+\beta_{1}x\right)+1\right)=1/\left(1+\exp\left(-\beta_{0}-\beta_{1}x\right)\right)$.

**Naive** **Bayes:** This is a simple probabilistic technique used as a classifier and based on the Bayes theorem with the naive assumption of conditional independence between every pair of features (independent variables) given the value of the class variable. NB is a conditional probability model for classification established by the independent variables $X=(X_{1},\ldots ,X_{p})$, which assigns the probabilities P(Y=c∣X=x) to each of *c* possible classes of *Y*. Therefore, by using the Bayes theorem, the conditional probability can be decomposed as P(Y=c∣X=x)=(P(Y=c)P(X=x∣Y=c))/P(X=x). Thus, the conditional probability model is derived, and then, the NB classifier is formed by this model and by the maximum value for some *c* stated as $\hat{y}={\mathrm{argmax}}_{c}\;P\left(Y=c\right)\prod_{j=1}^{p}P\left(X_{j}=x|Y=c\right)$, which indicates what class must be assigned to the new instance.

**Random** **forest:** This is an EM technique for regression or classification that allows us to construct multiple DT at training time and providing as the output the class corresponding to the mean prediction (regression) or the mode of the classes (classification) of the single trees. RF corrects using DT the possible overfitting in their training set. For example, RF works as follows if bootstrapping is used. Given a training set $x={\left(x_{1},\ldots ,x_{n}\right)}^{⊤}$ with responses variables $Y={\left(Y_{1},\ldots ,Y_{n}\right)}^{⊤}$, bagging repeatedly (for example, *B* times), select a random sample with the replacement of the training set, and fit DT to these samples as follows. For b=1,…,B: (i) sample, with replacement, *n* training sets from (x,Y), which are called ($x_{b},Y_{b}$); and (ii) train a classification or regression $f_{b}$ on ($x_{b},Y_{b}$). After training, predict for the testing set $x^{\prime }$ by averaging the predictions from all the single regressions on $x^{\prime }$, with $\hat{f}=\left(1/B\right)\sum_{b=1}^{B}f_{b}\left(x^{\prime }\right)$, or by taking the majority in the case of classification.

**Support** **vector** **machines:** This is a supervised technique associated with learning algorithms that analyze data employed for regression and classification. In the linear case, we must have a training set of *n* points of the form $\left(x_{1},Y_{1}\right),\ldots ,\left(x_{n},Y_{n}\right)$, where the $Y_{i}$ is either 1 or −1, each indicating the class to which the point $x_{i}$ belongs, with each $x_{i}$ being a *p*-dimensional vector. We want to find the maximum-margin hyperplane that separates the group of points $x_{i}$, when $Y_{i}=1$, from the group of points with $Y_{i}=-1$. This is defined so that the distance between the hyperplane and the nearest point $x_{i}$ from any group is maximized. Any hyperplane may be represented as the set of points $x_{i}$ that satisfy the condition w·x−b=0, where w is the normal vector to the hyperplane. The parameter b/∥w∥ determines the offset of the hyperplane from the origin along the normal vector w.

### 2.5. Data Mining/ML Algorithms’ Performance

**Figure 3:**
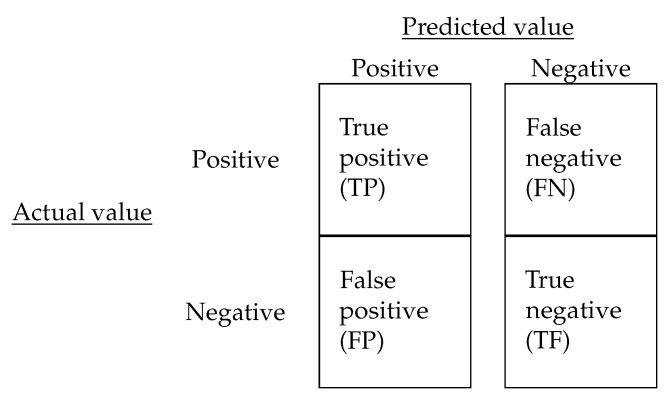
Confusion matrix and performance metrics.

The performance of the different ML algorithms considered can be evaluated using an *s*-fold cross-validation procedure, where the data set is split into *s* equal sets, s−1 of them used for training the model (training set), whereas the remaining set is used for testing the performance of the model (testing set) [46]. This procedure must be repeated a number of times (for example, s=10, one for each set), and the results may then be averaged for their evaluation ensuring that they are independent of the selected partition with respect to the training and testing sets. We use the performance metrics proposed in [47] to evaluate each ML algorithm considered. These metrics are described below and based on the confusion matrix presented in Figure 3.

**Accuracy:**  This represents a measure of the total number of instances correctly classified. For our case study, this considers both classes, students who are retained and those who drop out of their studies.

**TP** **rate:** For our case study, this is the proportion of retained students who are correctly predicted or classified by the learning algorithm, from all the retained students. We expect to keep this rate as high as possible, because it indicates the students who are retained and will continue studying.

**FP** **rate:** For our case study, this corresponds to the proportion of students who drop out, but the learning algorithm incorrectly classifies them as retained. We expect to maintain this rate as low as possible, because it indicates the students who will drop out and who the models will not detect.

**Precision:**  This is estimated as the ratio of the TP rate to the sum of the TP and FP rates, that is, precision = TP/(TP+ FP), indicating for our case study the proportion of retained students correctly classified from those who were predicted by the learning algorithm. We expected to maintain this precision as high as possible, because it indicates the correct number of students who will drop out between the number predicted as dropouts.

**F-measure:**  This is related to the harmonic mean of the precision and TP rate, a measure that ranges from zero to one, with a value close to one being considered as a good performance, because it indicates an equilibrium between precision and the TP rate.

**Root** **mean** **squared** **error** **(RMSE):** This is a measure of the differences between the values predicted by a model and the observed values. The RMSE is a measure of precision used to compare the prediction errors of different models for a particular data set. A small value of the RMSE means a better accuracy of the model.

**κ-statistic:**  This is a statistic that measures similarity or agreement. The κ-statistic is defined as $\kappa =\left(p_{a}-p_{e}\right)/\left(1-p_{e}\right)$, where $p_{a}$ is the proportion of times the raters agree, that is, the percentage of agreement between the classifier and ground truth; whereas $p_{e}$ is the proportion of times the raters are expected to agree by chance alone, that is, the chance of agreement. Values of the κ-statistic close to one indicate better results of the classifier.

**Friedman** **value** **(ranking):** The Friedman test is a non-parametric approach that is equivalent to the parametric one-way ANOVA test. Both tests are used to detect differences in multiple treatments. Given the data ${\left\{x_{ij}\right\}}_{n\times c}$, that is, a matrix with *n* rows (the replicates) and *c* columns (the treatments or classes), we calculate the ranks within each row. If ties existed, we assign to each tie the average of the ranks that would have been assigned without ties. The procedure is as follows: (i) replace the data with a new matrix ${\left\{r_{ij}\right\}}_{n\times c}$, where the entry $r_{ij}$ is the rank of $x_{ij}$ within row *i*; (ii) find the values ${\bar{r}}_{\cdot j}=\left(1/n\right)\sum_{i=1}^{n}r_{ij}$; (iii) calculate the test statistic given by $Q=\left(12n/\left(c(c+1)\right)\right)\sum_{j=1}^{c}{\left({\bar{r}}_{\cdot j}-\left(c+1\right)/2\right)}^{2}$. Observe that the value of *Q* needs to be adjusted for tied values in the data. When *n* or *c* is large (that is, n>15 or c>4), *Q* is approximately chi-squared distributed with c−1 degrees of freedom, and then, *p*-value $=P(\chi_{c-1}^{2}\geq Q)$. If *n* or *c* is small, the approximation to the chi-squared distribution is poor, and the *p*-value can be obtained from software. If the *p*-value is significant, suitable post-hoc multiple comparison tests must be performed.

### 2.6. Interpretation and Evaluation

In this final phase, the performance of different ML algorithms must be compared in order to select the best algorithm. One must focus on those algorithms that exhibit a high TP rate, precision, and κ-statistic, but a low FP rate and RMSE, as well as on those variables or features that seem to be more important to model the diverse scenarios considered in the study under analysis. Note that the main focus must be on the results of the IG analysis for the situations modeled, providing key features for identification. In addition, we apply the Friedman rank test [48] (pp. 262–274) to compare the different algorithms statistically [49]. For more details and tools about statistical tests for comparing the ML algorithms, the interested reader is referred to the web platform https://tec.citius.usc.es/stac (accessed on 11 April 2021), where one can verify the results obtained from the learning algorithms applying these statistics.

## 3. Case Study

In this section, we apply the methodology presented in Section 2 to the Chilean educational data and summarize in an algorithm this methodology.

### 3.1. ML Algorithms and Computer Configurations

In our study, the ML algorithms were trained to correctly distinguish among students who were retained from those who dropped out of the university, which is the focus of our study. We evaluate the ML algorithms: DT, KNN, LR, NB, RF, and SVM using the Weka software (V3.6.11) on a Windows 7 Professional 64-Bit, Intel Core i3-3120 Processor with 2.5 GHz and 10 GB of RAM; https://www.cs.waikato.ac.nz/ml/weka (accessed on 12 March 2015).

Algorithm 1 provides a summary of the methodology proposed in this study.
**Algorithm 1:** Methodology proposed to predict student retention/dropout in HE institutions similar to the Chilean case.1:Collect and input data about the PSU scores (or equivalent depending on the country), CP index, economic quintiles, and first/second year scores.2:Standardize attribute names in the input data.3:Perform cleanup and removal of missing/noisy/duplicate instances in input data.4:Eliminate data features that do not add value according to [16].5:Create a data subset using the PSU scores, CP index, and economic quintiles.6:Extract data of students with more than three years of academic follow-up.7:State classes for data as two values considering actives and dropouts.8:Arrange data for the global model from the full data set considering dropout at any level.9:Assemble data for the first level model from the full data set considering dropout in the first year only.10:Dispose data for the second-level model from the first-level data with the first-level retained students plus the first-year scores.11:Organize data for the third-level model from the second-level data with the second-level retained students plus the second-year scores.12:Apply ML algorithms to the global model with the data of Step 8, and analyze the results.13:Employ ML algorithms for the first-level model with the data of Step 9, and state the results.14:Use ML algorithms for the second-level model with the data of Step 10, and obtain the results.15:Utilize ML algorithms for third-level model with the data of Step 11, and indicate the results.16:Establish the performance of the ML algorithms, and propose the best one.

### 3.2. Data Selection

**Figure 4:**
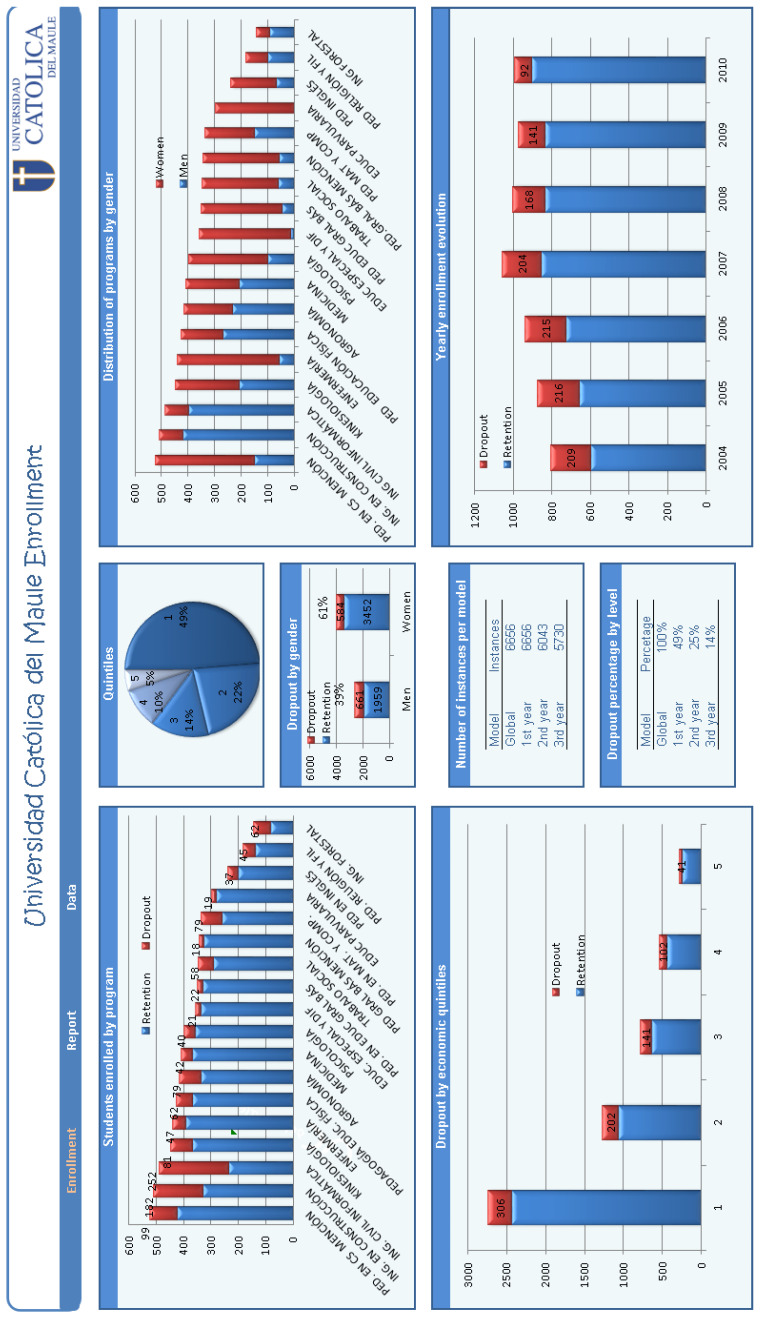
Dashboard of enrollment in the UCM.

As mentioned, the data used in this case study were obtained from UCM records. Until the year 2015, this university annually accepted approximately one-thousand freshman students, distributed through 18 undergraduate programs. As shown in the chart of the quintiles in Figure 4, nearly 50% of students enrolled at UCM came from the poorest national economic quintile, and only 5% belonged to the richest quintile. The data set selected for our study contains numerous variables related to demographic background, financial indicators, geographic origin, school performance, and university performance, among others. As also mentioned, the UCM imposed restrictions on the use of these data, when this study began in 2013, as they contain potentially identifiable or sensitive information about students. An agreement not to disclose these data was signed by the authors so that any request for them must be directed to its ethics committee (http://portal.ucm.cl/comite-etica-cientifico (accessed on 15 April 2021)).

Table 3 summarizes the most important variables and specific features considered at this stage of data selection. The study also considered a characteristic called the CP index, which was estimated for each student and related to the socioeconomic context of the place of origin from which the student came before entering the university. The list of features was then considered for this study after the pre-processing step and shown in Table 4.

At this stage, the data sources and types to be used were determined by extracting the most relevant data. These data considers existing records from different databases and various sources, including official data and statistics from the Chilean government (https://www.demre.cl [50] (accessed on 15 April 2021)), secondary educational institutions, and from the UCM databases. All these data were gathered in a warehouse composed of 6656 instances for students enrolled between the years 2004 and 2010. A total of 165 features were collected for each of the students involved in this study, including the target variable of interest. These features are the basis to predict the occurrence of dropout.

### 3.3. Preprocessing, Transformation of Data, and Initial Results

Construction of a data warehouse enables any university decision makers to obtain key performance factors for their organization, allowing them to query and deliver reports. In this study, we perform a cleanup and reduction of missing and/or noisy data, selecting the variables that provide the most relevant information. Initially, our data warehouse consisted of 165 variables, but after removing noisy data, forty-one variables were selected for further analysis; see Section 2.3.

As described in Figure 1, four predictive models are evaluated in this research. The first one is a global model for the prediction of student retention regardless of the level (year) when student dropout occurs. The second model attempts to predict student retention for those who dropped their studies during the first year. The third and fourth models are used to predict student retention during the second and third year of study, respectively. The first and second models utilize 21 variables from the total set selected, because university grades are not employed in these cases. Instead, our study considers variables related to the data of students prior to enrollment to their university. For the third and fourth model, thirty-one and 41 variables are utilized, respectively. The academic records of students considering their educational performance data during the first and second year of study are incorporated into the modeling.

An IG procedure is developed to rank the variables used in each scenario modeled. Table 5 exhibits the variable ranking by using the IG index for the four models evaluated, considering the first 20 variables with the highest IG scores in each case. Among the best-ranked variables, secondary school academic performance is particularly relevant. Demonstrated by the first rank position of NEM, this variable represents the average for secondary school grades, obtained in the first three scenarios modeled. However, this variable does not appear in the ranking related to the fourth model. Note that, in general, other variables associated with the PSU scores are not highly ranked in the models. Interestingly, the CP index is in the second position of the IG rankings for the global, first-, and second-level models. Various reasons for this lack of importance can be postulated. Perhaps the CP index weighs more on early dropout. Similar to the case of the NEM variable, it seems not to be pertinent for the fourth model. The relevance of the CP index for the prediction of university student retention has not been reported in previous studies. Nevertheless, this factor seems to have a high influence and should be studied further. Different from the situation addressed in [51], in our study, the information from the economic quintile seems to be less relevant, and it is classified from the sixth position for the majority of the models analyzed. In addition, the variables associated with university student performance, which are incorporated only for the third and fourth models, have a relevant position in the ranking. Therefore, the fourth model describes the positive values of the IG indicator, which are much less relevant in comparison to the previous models. This indicator shows that the task associated with predicting student retention at this level is a more difficult problem and that dropout may be affected by factors not included here.

### 3.4. Performance Evaluation of Predictive Models

Our study focused on the prediction of students who dropped out of the university, considering different scenarios depending on the period when they effectively dropped out of the university. Four different situations were established for modeling as schematized in Figure 1. The defined problem comprised two classes: (i) the positive class associated with students who were retained by the university and (ii) the negative class associated with students who dropped out of the university at different levels. These retention and dropout issues are common in the world. Thus, this study has relevance for HE institutions around the world with similar conditions to the Chilean case. Several ML algorithms were trained so that they aimed to classify or predict whether a student who entered the university would be successful (that is, she/he will finish their studies) or will drop out of her/his studies during the process. The performance of the predictive models were evaluated for the different model scenarios, focused on those exhibiting a high TP rate, low FP rate, low RMSE, and a κ-statistic close to one. High values of the accuracy, precision, and F-measure were also employed for evaluating precision and accuracy. In addition, as mentioned, we applied the Friedman test for comparing the different algorithms, arriving at results similar to those obtained with the other measures.

**Global** **model** **results:** The global model involves the prediction of all students, regardless of the level when they dropped out of the university. Table 6 reports the performance of the different ML algorithms evaluated. Most of the assessed learning algorithms exhibited accuracies near or slightly over 80%. In addition, most models evaluated presented high TP rates with values ranging from 0.89 to 0.98, which indicated good performance, mainly on the prediction of retained students. However, at the same time, high values for the FP rates over 0.63 were observed for all the learning algorithms evaluated. Nevertheless, for all models, the RMSE exceeded 0.36, and the κ-statistics were low, being less than 0.28. These results imply that ML algorithms show difficulties in correctly predicting students who drop out of their programs, as also reported in [7,8]. This situation could reveal a problem associated with an unbalanced class, in which the number of instances of the majority class (that is, retained students) exceeds by five times the number of instances in the minority dropout class. Thus, the learning algorithms tend to bias their results towards the prediction of instances in the majority class. To address the unbalanced class problem, we followed a methodology named the synthetic minority over-sampling technique (SMOTE) [52]. In this technique, instances of the classes are artificially balanced, increasing the number of instances in the minority class, in order to decrease the effect on the performance of the learning algorithms. SMOTE is an accepted methodology to deal with unbalanced class situations.

Table 7 reports the performance of different ML algorithms evaluated under the balanced class situation. We use 10-fold cross-validation to perform the training and testing process for prediction. The accuracy, as well as the values for the precision and F-measure scores, remained high for all models. The effect of balancing the classes was clearly noticeable because of an important reduction of the FP rates ranging from 0.13 to 0.22 for all learning algorithms evaluated, while maintaining relatively high scores for the TP rates, with a slight decrease in the RMSE and an increase in the value of the κ-statistic, now being between 0.58 and 0.68. Among the ML techniques evaluated, the performance obtained by the RF technique, reaching good values in all performance measures considered, outperformed the other techniques. Even though the best performance for the FP rate was obtained by the KNN technique, this also exhibited low scores for the TP rate, accuracy, and F-measure compared to the RF technique. The results obtained from the Friedman test for the ML algorithms are also reported in Table 7. According to this table, both the RF and SVM algorithms ranked first based on the post-hoc multiple comparisons established between all the ML algorithms; see Appendix A (Table A1). The statistical results of the Friedman rank test presented in Table 7 confirmed that the superiority of the RF algorithm over the four ML algorithms analyzed was not random.

**First-level** **model** **results:** The first-level model was focused only on assessing freshman dropout. This case also faced the unbalanced class situation as stated in the case of the global model. Here, the relationship between the instances of majority and minority classes was 11-1 and with the same problems related to the prediction of the minority class. SMOTE was again employed in order to balance the classes. The performance of different ML algorithms is exhibited on Table 8. An important increment occurred in the accuracy of all the learning algorithms evaluated, reaching values near 90%. This was coincident with an increment in the TP rate, a reduction in the FP rate, a decrease in the RMSE, and an increase in the value of the κ-statistic, for most cases. In the first-level model scenario, the RF technique also exhibited the best performance among techniques on all the measures calculated, reaching the lowest FP rate (0.119) and RMSE (0.238), as well as the highest TP rate (0.976) and κ-statistic (0.868). The KNN technique also improved importantly its performance compared to the global model scenario. The results reported with the Friedman test for the ML algorithms are also in Table 8. From this table, the RF algorithm ranked first, and the DT algorithm was in second position. According to this table, both the RF and DT algorithms ranked first based on the post-hoc multiple comparisons established between all the ML algorithms; see Appendix A (Table A2). Once again, the statistical results of the Friedman rank test presented in Table 8 confirmed that the superiority of the RF algorithm was not random.

**Second-level** **model** **results:** The second-level model, which considered dropout for second-year students, provided information not included in the previous two models related to academic performance during the first year of HE institutions. The previous relationship to the balance of classes was 19-1 in this case. SMOTE was also employed here in order to balance the classes, as again the algorithms showed problems when predicting the minority class instances. The performance of different ML algorithms is exhibited in Table 9. In this case, an increment occurred in the accuracy and TP rates for most learning algorithms evaluated, compared to those obtained in the global and first-level model scenarios. However, at the same time, an increment occurred in the FP rate compared to previous model scenarios. Thus, in this second-level model scenario, SMOTE was unable to completely cope with the unbalanced class situation, and learning algorithms seemed to reduce their capacity to efficiently predict dropout students. Among the learning algorithms evaluated in this model scenario, there was no important difference between the performances of the RF and KNN techniques. The results of the Friedman test for the ML algorithms presented in Table 9 indicated once again that the RF algorithm was superior. However, the other ML algorithms were closely positioned with only the NB algorithm being statistically different from the RF algorithm based on the post-hoc multiple comparisons established between all the ML algorithms; see Appendix A (Table A3). Once again, the statistical results of the Friedman rank test presented in Table 9 confirmed that the superiority of the RF algorithm was not random.

**Third-level** **model** **results:** Similar to the previous model scenarios, an unbalanced class situation existed, in which the relationship between classes was 33-1. The performance of ML algorithms after applying SMOTE is exhibited in Table 10. In this case, the FP rates of most algorithms evaluated had an important increment, reaching similar values to those obtained when no balancing by SMOTE was utilized. This clearly indicates that SMOTE was unable to adequately reduce the unbalanced class situation. However, the result obtained by the NB technique was interesting, because it remained better than the other algorithms and still performed well enough. The results obtained from the Friedman test for the ML algorithms are also reported in Table 10. According to this table, both the RF and DT algorithms ranked first based on the post-hoc multiple comparisons established between all the ML algorithms; see Appendix A (Table A4). The statistical results of the Friedman rank test presented in Table 10 confirmed that the superiority of the RF algorithm over the four ML algorithms analyzed was not random.

### 3.5. Interpretation and Evaluation

With the results obtained in this case study, it is clear that predictive models at various levels can be applied to prevent dropout in any HE institution around the world with similar conditions to the Chilean case. In the present case study, we gave new insights and obtained a deeper understanding of the student retention/dropout problem in the Chilean HE institutions, based on the case of UCM. In addition, testing the predictive model as universally as applicable can aid in the design of alternatives and strategies for decision-makers to reduce student dropout rates in their institutions.

## 4. Conclusions, Results, Limitations, Knowledge Discovery, and Future Work

In this study, we focused on two aspects: (i) the use of data mining and machine learning algorithms to extract information; and (ii) the utilization of this information for knowledge discovery in databases. When using such algorithms, we formulated models based on them to predict student retention at three levels of study, employing higher education data and specifying the relevant variables involved in the modeling. Then, once the machine learning algorithms were applied and the final models were obtained, knowledge discovery could be extracted for higher education student retention based on a case study in Chile.

Regarding the data mining and machine learning algorithms, the results obtained with the predictive models formulated in this research indicated that predicting student dropout was possible at an accuracy that exceeded 80% in most scenarios and reaching false positive rates ranging from 10% to 15% in most cases; see Table 6, Table 7, Table 8, Table 9 and Table 10. Regardless of the machine learning algorithm used, in all evaluated scenarios, it was necessary to balance the classes. This procedure generated better results for the first three models (global, first-level, and second-level). However, the third-level model did not have good predictive performance, likely due to the excessive difference between the number of instances utilized to train the learning models, which was a limitation of our study. Among all the machine learning algorithms evaluated, the random forest technique performed best in general, especially when comparing in terms of false positive and true positive rates, precision, F-measure, root mean squared error, and κ-statistic. The random forest technique is robust, simple to understand, and implements logic diagrams, which can be used during its construction. Furthermore, the Friedman rank test was applied in order to further analyze the performance of the ML algorithms. Based on the results of this statistical analysis, it was found that the random forest algorithm ranked first among the studied algorithms, and its superiority was not random. Therefore, general analysis of the statistical results confirmed the superiority of the random forest algorithm, making it more competitive than the other five analyzed algorithms.

Regarding the knowledge discovery in databases, this study enabled the design of educational data mining alternatives for decision-makers in order to reduce dropout rates. Thus, our study exposed the benefits of a methodology useful to predict dropout for each higher education institution supplying data, as well as to demonstrate credit risk for banking institutions or government programs, providing financial assistance to higher education applicants. Therefore, students who are at risk of dropout can be identified prior to beginning their studies, proposing actions that can be directed towards at-risk students, thereby minimizing attrition and directing retention resources only to targeted students. The models in this case study predicted the level (year) when the student would drop out, which enables further focusing resources allocated to reducing dropout rates. For students who drop out at the end of the first year, the university will no longer receive the income corresponding to four years. Thus, for every student retained, the university could increase its annual income each year during the career program (4–5 years). From the viewpoint of students, the opportunity cost of studying instead of working is also high. For the Chilean government and other organizations, retention and dropout are similarly relevant. In our case study, over 70% of Chilean higher education students were from low economic quintiles. Thus, this information should be considered when assigning loans that are guaranteed by the government for education that is not completed. We detected that secondary education grades and the community poverty index were important predictive variables, which have not been previously reported in educational studies of this type. The dropout assessment at various levels reported here is valid for higher education institutions around the world with similar conditions to the Chilean case, where dropout rates affect the efficiency of such institutions. Having the ability to predict dropout based on student’s data enables these institutions to take preventative measures, avoiding the dropout.

We propose the following plans/recommendations to be developed:

(a) Implement a new information system that enables different databases to coexist for the quick acquisition of necessary information. Data warehouse compilation requires extensive time to extract the relevant data from university records.
(b) Establish a data-monitoring plan to track the enrollment of all students for further analysis and decision-making.
(c) Create a model for predicting students at risk of dropout at different levels of study.
(d) Employ a welcome plan for at-risk students who are identified by the predictive model, in order to assist in improving academic results.
(e) Offer a support program at all grade levels for identifying at-risk students.
(f) In order to increase the innovation of future works, a voting scheme of the machine learning algorithms used can be proposed or the explainability of an examined classifier may be promoted. Voting is an ensemble learning algorithm that, for example in regression, performs a prediction from the mean of several other regressions. In particular, majority voting is used when every model carries out a prediction (votes) for each test instance and the final output prediction obtains more than half of the votes. If none of the predictions reach this majority of votes, the ensemble algorithm is not able to perform a stable prediction for such an instance.

The authors are considering future research on modifications to the existing methodology about educational data and of other types in order to improve its accuracy.

## Figures and Tables

**Table 1:** Abbreviations, acronyms, notations, and symbols employed in the present document.

Abbreviations/Acronyms		Notations/Symbols	
ANN	artificial neural networks	∼	distributed as
CLU	clustering	*k*	number of nearest neighbors
CP	community poverty index	*n*	sample size
DT	decision trees	$l=\beta_{0}+\beta_{1}x$	log-odd
EDM	educational data mining	$o=b^{\beta_{0}+\beta_{1}x}$	odd
EM	ensemble models	$\beta_{0},\beta_{1}$	regression coefficients
FN	false negative	*X*	independent variable or feature
FP	false positive	*Y*	dependent variable or response
HE	higher education	p=P(Y=1)	probability function of LR
IG	information gain	$\;=\frac{\exp(\beta_{0}+\beta_{1}x)}{\exp(\beta_{0}+\beta_{1}x)+1}$	
KNN	*k*-nearest neighbors	$\;=\frac{1}{1+\exp(-\beta_{0}-\beta_{1}x)}$	
LR	logistic regression	P(Y=c∣X=x)	probability *Y* given X
ML	machine learning	$\frac{P(Y=c)P(X=x|Y=c)}{P(X=x)}$	Bayes conditional probability
NB	naive Bayes	$X=(X_{1},\ldots ,X_{p})$	vector of independent variables
NEM	secondary educational score	$\left(x_{1},Y_{1}\right),\cdots ,\left(x_{n},Y_{n}\right)$	instances
	(notas enseñanza media)	*c*	number of classes
PSU	university selection test	∥x∥	norm of a point *x*
	(prueba selección universitaria)	*s*	number of folds in cross-validation
RAM	random access memory	w	normal vector to the hyperplane
RF	random forest	TP/(TP + FP)	precision
SVM	support vector machines	$\kappa =\left(p_{a}-p_{e}\right)/\left(1-p_{e}\right)$	κ-statistic
TF	true negative	$p_{a}$	% of agreement classifier/ground truth
TP	true positive	$p_{e}$	agreement chance
UCM	Catholic University of Maule	$Q=\frac{12n}{c(c+1)}\sum_{j=1}^{c}{\left({\bar{r}}_{\cdot j}-\frac{c+1}{2}\right)}^{2}$	Friedman statistic
	(Universidad Católica del Maule)	${\left\{x_{ij}\right\}}_{n\times c}$	n×c data matrix
SMOTE	synthetic minority	${\left\{r_{ij}\right\}}_{n\times c}$	n×c rank matrix
	over-sampling technique	${\bar{r}}_{\cdot j}=\frac{1}{n}\sum_{i=1}^{n}r_{ij}$	rank average of column *j*
KDD	knowledge discovery	$P\left(\chi_{c}^{2}\geq Q\right)$	*p*-value
	in databases	$\chi_{c}^{2}$	chi-squared distribution
			with *c* degrees of freedom

**Table 2:** Comparison of data mining techniques in the literature to predict university student retention.

Reference	Instances	Technique(s)	Confusion Matrix	Accuracy	Institution	Country
[7,8]	16,066	ANN, DT, SVM, LR	Yes	87.23%	Oklahoma State	
					University	USA
[23]	713	DT, NB, LR, EM, RF	Yes	80%	Eindhoven University	
					of Technology	Netherlands
[24]	N/A	ANN, SVM, EM	No	N/A	National Technical	
					University of Athens	Greece
[25]	8025	DT, NB	Yes	79%	Kent State	
					University	USA
[26]	452	ANN, DT, KNN	Yes	N/A	University	
					of Chile	Chile
[27]	6078	NN, NB	Yes	N/A	Roma Tre	
					University	Italy
[28]	17,910	RF, DT	Yes	N/A	University	
					of Duisburg	Germany
[29]	N/A	LR, DT, ANN, EM	No	N/A	N/A	
					N/A	USA
[30]	1500	CLU, SVM, RF	No	N/A	University	
					of Bologna	Italy
[31]	6470	DT	No	87%	Mugla Sitki	
					Kocman University	Turkey
[32]	811	EM, NB, KNN, ANN	No	N/A	Mae Fah	
					Luang University	Thailand
[33]	3877	LR, SVM, DT	No	N/A	Purdue	
					University	USA
[34]	456	ANN, DT	No	N/A	University of	
					Computer Science	Cuba
[35]	1359	NB, SVM	Yes	87%	Federal University	
					of Rio de Janeiro	Brazil
[36]	N/A	N/A	No	61%	Unitec Institute	New
					of Technology	Zealand
[37]	22,099	LR, DT, ANN	No	N/A	several	
					universities	USA
[38]	1055	C45, RF, CART, SVM	No	86.6%	University	
					of Oviedo	Spain
[39]	6500	DT, KNN	No	98.98%	Technical University	
					of Izúcar	Mexico
[40]	N/A	DT	Yes	N/A	N/A	
					N/A	India
[41]	6690	ANN, LR, DT	No	76.95%	Arizona State	
					University	USA

**Table 3:** Most representative variables considered for the UCM case study.

Attributes	Features
Demographic background	Name, age, gender.
Geographic origin	Place of origin, province.
Socioeconomic index	CP index.
School performance	High school grades, secondary educational score (NEM), PSU score.
University performance	Number of approved courses, failed courses, approved credits, failed credits.
Financial indicators	Economic quintile, family income.
Others	Readmissions, program, application preference, selected/waiting list, health insurance.

**Table 4:** List of variables selected after the pre-processing step for the UCM case study.

Attributes		
Age Application preference Approved credits 1th semester Approved credits 2nd semester Approved credits 3rd semester Approved credits 4th semester Approved courses 1th semester Approved courses 2nd semester Approved courses 3rd semester Approved courses 4th semester CP index Dependent group Educational area Entered credits 1th semester	Entered credits 2nd semester Entered credits 3rd semester Entered credits 4th semester Family income Gender Graduate/non-graduate Health insurance Marks 1th semester Marks 2nd semester Marks 3rd semester Marks 4th semester NEM Program Province	PSU averaged score in language/maths PSU score of language PSU score of maths PSU score of specific topic PSU weighted score Quintile Readmissions Registered courses 1th semester Registered courses 2nd semester Registered courses 3rd semester Registered courses 4th semester School Selected/waiting list

**Table 5:** Ranking of features using the IG procedure for each model scenario in the UCM case study.

		Global		First Level		Second Level		Third Level
Rank	IG	Variable	IG	Variable	IG	Variable	IG	Variable
1	0.430	NEM	0.511	NEM	0.357	NEM	0.098	Marks 3rd semester
2	0.385	CP index	0.468	CP index	0.220	CP index	0.087	Marks 4th semester
3	0.209	Program	0.286	School	0.211	School	0.084	Approved courses 3rd semester
4	0.204	School	0.190	Program	0.211	Approved courses 2nd semester	0.083	Approved courses 2nd semester
5	0.105	PSU specific topic	0.112	PSU specific topic	0.195	Approved credits 2nd semester	0.074	School
6	0.068	Quintile	0.110	PSU language	0.183	Approved credits 1st semester	0.069	Marks 1st semester
7	0.059	Gender	0.098	Quintile	0.176	Approved courses 1st semester	0.067	Approved courses 4th semester
8	0.051	Family income	0.056	Age	0.163	Marks 1st semester	0.066	Approved courses 1st semester
9	0.041	Age	0.053	Educational area	0.149	Program	0.063	Marks 2nd semester
10	0.037	Educational area	0.047	PSU weighted score	0.141	Marks 2nd semester	0.059	Approved credits 1st semester
11	0.034	PSU language	0.043	Graduate/non-graduate	0.130	Entered credits 2nd semester	0.059	Entered credits 2nd semester
12	0.030	Province	0.037	Family income	0.103	Entered credits 1st semester	0.056	Approved credits 2nd semester
13	0.027	Application preference	0.034	Province	0.079	Registered courses 2nd semester	0.051	Approved credits 4th semester
14	0.026	Health insurance	0.033	Gender	0.058	Gender	0.049	Entered credits 3rd semester
15	0.025	Readmissions	0.030	PSU math	0.038	Registered courses 1st semester	0.049	Program
16	0.025	PSU weighted score	0.029	Readmissions	0.032	Province	0.048	Entered credits 4th semester
17	0.019	PSU math	0.028	Health insurance	0.030	Family income	0.044	Approved credits 3rd semester
18	0.015	Graduate/non-graduate	0.025	PSU language/math	0.029	Quintile	0.042	Registered courses 1st semester
19	0.014	PSU language/math	0.022	Application preference	0.025	Age	0.030	Registered courses 3rd semester
20	0.001	Dependent group	0.001	Dependent group	0.024	Educational area	0.030	Registered courses 4th semester

**Table 6:** Global model performance considering an unbalanced class situation for the UCM case study.

ML Algorithm	Accuracy	Precision	TP Rate	FP Rate	F-Measure	RMSE	κ-Statistic
DT	82.75%	0.840	0.973	0.806	0.902	0.365	0.227
KNN	81.36%	0.822	0.984	0.929	0.896	0.390	0.082
LR	82.42%	0.849	0.954	0.739	0.898	0.373	0.271
NB	79.63%	0.860	0.894	0.631	0.877	0.387	0.283
RF	81.82%	0.829	0.979	0.879	0.897	0.370	0.143
SVM	81.67%	0.828	0.977	0.881	0.897	0.428	0.138

**Table 7:** Global model performance considering a balanced class situation for the UCM case study.

Algorithm	Accuracy	Precision	TP Rate	FP Rate	F-Measure	RMSE	κ-Statistic	Friedman Value (Ranking)
DT	82.19%	0.814	0.837	0.194	0.825	0.368	0.644	3.49475 (4)
KNN	83.93%	0.859	0.814	0.135	0.836	0.363	0.679	3.61317 (6)
LR	83.45%	0.825	0.851	0.182	0.838	0.351	0.669	3.48317 (3)
NB	79.14%	0.791	0.796	0.213	0.793	0.399	0.583	3.51025 (5)
RF	88.43%	0.860	0.920	0.151	0.889	0.301	0.769	3.45125 (1)
SVM	83.97%	0.822	0.869	0.190	0.845	0.400	0.679	3.44875 (1)

**Table 8:** First-level model performance for the UCM case study.

Algorithm	Accuracy	Precision	TP Rate	FP Rate	F-Measure	RMSE	κ-Statistic	Friedman Value (Ranking)
DT	89.21%	0.888	0.933	0.166	0.910	0.294	0.775	3.44033 (1)
KNN	89.43%	0.929	0.887	0.096	0.908	0.298	0.784	3.61133 (6)
LR	87.70%	0.885	0.908	0.166	0.896	0.309	0.745	3.48183 (4)
NB	83.95%	0.869	0.854	0.181	0.862	0.349	0.671	3.56733 (5)
RF	93.65%	0.921	0.976	0.119	0.947	0.238	0.868	3.42083 (1)
SVM	88.30%	0.889	0.914	0.160	0.901	0.342	0.758	3.47883 (3)

**Table 9:** Second-level model results for the UCM case study.

ML Algorithm	Accuracy	Precision	TP Rate	FP Rate	F-Measure	RMSE	κ-Statistic	Friedman Value (Ranking)
DT	91.06%	0.938	0.954	0.288	0.946	0.278	0.687	3.45675 (3)
KNN	94.41%	0.965	0.967	0.161	0.966	0.222	0.809	3.49425 (5)
LR	93.57%	0.958	0.964	0.193	0.961	0.232	0.779	3.48625 (4)
NB	86.69%	0.954	0.880	0.194	0.916	0.347	0.603	3.69075 (6)
RF	95.76%	0.959	0.99	0.196	0.975	0.193	0.847	3.41825 (1)
SVM	94.40%	0.958	0.975	0.196	0.966	0.237	0.804	3.45425 (2)

**Table 10:** Third-level model performance for the UCM case study.

ML Algorithm	Accuracy	Precision	TP Rate	FP Rate	F-Measure	RMSE	κ-Statistic	Friedman Value (Ranking)
DT	94.99%	0.955	0.993	0.739	0.974	0.208	0.360	3.35866 (1)
KNN	96.90%	0.977	0.990	0.371	0.984	0.168	0.689	3.43132 (3)
LR	90.58%	0.973	0.926	0.414	0.949	0.305	0.376	3.60561 (5)
NB	88.09%	0.987	0.885	0.181	0.933	0.331	0.396	3.76270 (6)
RF	96.92%	0.969	0.999	0.503	0.984	0.160	0.641	3.38406 (1)
SVM	96.17%	0.978	0.982	0.356	0.980	0.196	0.644	3.45825 (4)

## Data Availability

Data cannot be shared publicly as they contain potentially identifiable or confidential student information. Result and computational procedures are available upon request from the authors. The authors declare to honor the Principles of Transparency and Best Practice in Scholarly Publishing about Data.

## Appendix A. Friedman Test Results and Post-Hoc Analysis

**Table A1:** Friedman test results and post-hoc analysis for the global model.

Friedman Test (Significance Level of 0.05)			
Statistic	*p*-value	Result	
360.428080	0.00000	H0 is rejected	
Friedman Value	Algorithm	Ranking	
3.44875	SVM	1	
3.45125	RF	2	
3.48317	LR	3	
3.49475	DT	4	
3.51025	NB	5	
3.61317	KNN	6	
**Post-Hoc Analysis (Significance Level of 0.05)**			
Comparison	Statistic	*p*-value	Result
KNN vs. SVM	4.16872	0.00046	H0 is rejected
KNN vs. RF	4.10534	0.00057	H0 is rejected
KNN vs. LR	3.29610	0.01274	H0 is rejected
KNN vs. DT	3.00241	0.03214	H0 is rejected
KNN vs. NB	2.60941	0.09977	H0 is accepted
NB vs. SVM	1.55931	1.00000	H0 is accepted
NB vs. RF	1.49592	1.00000	H0 is accepted
DT vs. SVM	1.16631	1.00000	H0 is accepted
RF vs. DT	1.10293	1.00000	H0 is accepted
LR vs. SVM	0.87262	1.00000	H0 is accepted
RF vs. LR	0.80924	1.00000	H0 is accepted
NB vs. LR	0.68669	1.00000	H0 is accepted
NB vs. DT	0.39300	1.00000	H0 is accepted
LR vs. DT	0.29369	1.00000	H0 is accepted
RF vs. SVM	0.06339	1.00000	H0 is accepted

**Table A2:** Friedman test results and post-hoc analysis for the first-level model.

Friedman Test (Significance Level of 0.05)			
Statistic	*p*-value	Result	
361.260066	0.00000	H0 is rejected	
Friedman Value	Algorithm	Ranking	
3.42083	RF	1	
3.44033	DT	2	
3.47883	SVR	3	
3.48183	LR	4	
3.56733	NB	5	
3.61133	KNN	6	
**Post-Hoc Analysis (Significance Level of 0.05)**			
Comparison	Statistic	*p*-value	Result
KNN vs. RF	4.83006	0.00002	H0 is rejected
KNN vs. DT	4.33564	0.00020	H0 is rejected
NB vs. RF	3.71445	0.00265	H0 is rejected
KNN vs. SVR	3.35949	0.00937	H0 is rejected
KNN vs. LR	3.28342	0.01128	H0 is rejected
NB vs. DT	3.22004	0.01282	H0 is rejected
NB vs. SVR	2.24388	0.22356	H0 is accepted
NB vs. LR	2.16782	0.24138	H0 is accepted
RF vs. LR	1.54663	0.85366	H0 is accepted
RF vs. SVR	1.47057	0.85366	H0 is accepted
KNN vs. NB	1.11560	1.00000	H0 is accepted
LR vs. DT	1.05222	1.00000	H0 is accepted
DT vs. SVR	0.97615	1.00000	H0 is accepted
RF vs. DT	0.49442	1.00000	H0 is accepted
LR vs. SVR	0.07606	1.00000	H0 is accepted

**Table A3:** Friedman test results and post-hoc analysis for the second-level model.

Friedman Test (Significance Level of 0.05)			
Statistic	*p*-value	Result	
362.345869	0.00000	H0 is rejected	
Friedman Value	Algorithm	Ranking	
3.41825	RF	1	
3.45425	SVM	2	
3.45675	DT	3	
3.48625	LR	4	
3.49425	KNN	5	
3.69075	NB	6	
**Post-Hoc Analysis (Significance Level of 0.05)**			
Comparison	Statistic	*p*-value	Result
NB vs. RF	6.90914	0.00000	H0 is rejected
SVM vs. NB	5.99637	0.00000	H0 is rejected
NB vs. DT	5.93298	0.00000	H0 is rejected
NB vs. LR	5.18502	0.00000	H0 is rejected
KNN vs. NB	4.98218	0.00001	H0 is rejected
KNN vs. RF	1.92695	0.53986	H0 is accepted
RF vs. LR	1.72411	0.76218	H0 is accepted
KNN vs. SVM	1.01419	1.00000	H0 is accepted
RF vs. DT	0.97615	1.00000	H0 is accepted
KNN vs. DT	0.95080	1.00000	H0 is accepted
SVM vs. RF	0.91277	1.00000	H0 is accepted
SVM vs. LR	0.81135	1.00000	H0 is accepted
LR vs. DT	0.74796	1.00000	H0 is accepted
KNN vs. LR	0.20284	1.00000	H0 is accepted
SVM vs. DT	0.06339	1.00000	H0 is accepted

**Table A4:** Friedman test results and post-hoc analysis for the third-level model.

Friedman Test (Significance Level of 0.05)			
Statistic	*p*-value	Result	
360.476685	0.00000	H0 is rejected	
Friedman Value	Algorithm	Ranking	
3.35866	DT	1	
3.38406	RF	2	
3.43132	KNN	3	
3.45825	SVM	4	
3.60561	LR	5	
3.76270	NB	6	
**Post-Hoc Analysis (Significance Level of 0.05)**			
Comparison	Statistic	Adjusted *p*-value	Result
KNN vs. NB	8.33467	0.00000	H0 is rejected
NB vs. RF	9.52320	0.00000	H0 is rejected
NB vs. DT	10.16220	0.00000	H0 is rejected
SVM vs. NB	7,65733	0.00000	H0 is rejected
LR vs. DT	6.21106	0.00000	H0 is rejected
RF vs. LR	5.57207	0.00000	H0 is rejected
KNN vs. LR	4.38353	0.00011	H0 is rejected
NB vs. LR	3.95114	0.00062	H0 is rejected
SVM vs. LR	3.70619	0.00147	H0 is rejected
SVM vs. DT	2.50487	0.07350	H0 is accepted
SVM vs. RF	1.86588	0.31029	H0 is accepted
KNN vs. DT	1.82754	0.31029	H0 is accepted
KNN vs. RF	1.18854	0.70387	H0 is accepted
KNN vs. SVM	0.67734	0.99638	H0 is accepted
RF vs. DT	0.63900	0.99638	H0 is accepted
